# Torsion

**Published:** 1872-03

**Authors:** 


*Torsion.*—In a letter to the *Missouri Dental Journal*, entitled "A few Days Among the European Dentists," Dr. E. A. Bogue, of New York, describes the following operation:

"At Mr. Sercombe's I was fortunate enough to witness a case of torsion, about which we have heard so much and seen so little that was favorable. In this case the patient, a little boy of twelve years or thereabouts, was anæsthetized by nitrous oxide, and one of the central incisors, which was about three-fourths erupted, in a position nearly at right angles with what it should have been, was operated on. This incisor was seized with a pair of fat-nosed pliers, armed with buckskin so as to prevent injury to the tooth, pressed strongly UP, and, while so pressed up, gradually rotated back and forth until the tooth was felt to yield in the socket, when it was slowly turned to a little beyond the position to which it was intended to occupy, and a retaining plate was placed in the mouth.

This retaining plate had been previously prepared by cutting the plaster tooth from a cast of the mouth, placing it in the desired position, and on that, vulcanizing the plate. This completed the operation; and I could not help a contrast most unfavorable to ourselves, as I recollected the long and tedious processes resorted to among us for the accomplishment of the same end, and the frequent ill success attending even them.

Remarking upon what might be hoped for in the future of this case, Mr. Sercombe kindly gave me an opportunity to see two other cases, one of which had been done about two years previously, and I was obliged to confess the success was most pleasing in every respect—pulps living, teeth in position, one of them affected by a slight recession of the gum, not noticeable except to the professional eye, but further than that no trace of what had been a marked deformity.

On a subsequent occasion, speaking with Mr. Tomes about the operation, he said that he knew of no cases in which the patients were young, where the pulps had been destroyed, or other perceptible injury had supervened, although they had treated quite a large number at the dental hospital within a few years past, and he thought if proper care were used there was no physiological reason against the operation."

